# The Effects of Synthetic Estrogen Exposure on the Sexually Dimorphic Liver Transcriptome of the Sex-Role-Reversed Gulf Pipefish

**DOI:** 10.1371/journal.pone.0139401

**Published:** 2015-10-08

**Authors:** Emily Rose, Sarah P. Flanagan, Adam G. Jones

**Affiliations:** Department of Biology, Texas A&M University, 3258 TAMU, College Station, Texas, 77845, United States of America; John Hopkins University School of Medicine, UNITED STATES

## Abstract

Species exhibiting sex-role reversal provide an unusual perspective on the evolution of sex roles and sex differences. However, the proximate effects of sex-role reversal are largely unknown. Endocrine disruptors provide an experimental mechanism to address hormonal regulation of sexually dimorphic gene expression in sex-role-reversed taxa. Here, we investigate gene expression patterns in the liver of the sex-role-reversed Gulf pipefish, because the liver is known to be sexually dimorphic and estrogen-regulated in species with conventional sex roles. Using next-generation RNA-sequencing technology (RNA-seq), we detected sexually dimorphic hepatic gene expression patterns, with a total of 482 differentially expressed genes between the sexes in Gulf pipefish. Two-thirds of these genes were over-expressed in females, and the sex-specific transcriptomes of this sex-role-reversed pipefish’s liver were superficially similar to those of fishes with conventional sex-roles. We exposed females, pregnant males, and non-pregnant males to 17α-ethinylestradiol (EE2) at ecologically relevant concentrations of 5ng/L and compared gene expression patterns in the livers of exposed fish to control fish. Several genes that were up-regulated in EE2-exposed males relative to control males were also found to be female-biased in control animals. These genes included several of the classic estrogen biomarkers, such as *vitellogenin*, *choriogenin*, and *zona pellucida*. Thus, estrogen exposure induced feminization of the male liver transcriptome in a sex-role-reversed pipefish. These results suggest that the ancestral state of estrogen-regulated female reproductive physiology has been retained in all sex-role-reversed vertebrates thus far studied, despite substantial evolution of the hormonal regulation of ornamentation and mating behavior in these interesting taxa.

## Introduction

Sex-role-reversed species, in which females tend to evolve elaborate secondary sexual traits and males tend to be choosy, have enjoyed a rich history in the study of the evolution of sex differences and sex roles. In the sexual selection literature, sex-role reversal is usually defined as the situation in which sexual selection acts more strongly on females than on males [1], and it is normally associated with substantial parental investment by males [2], which reduces the male potential reproductive rate below that of the female [3,4]. Sex-role-reversed species have provided unique opportunities to study sexual selection, because they have allowed novel tests of hypotheses related to mating competition and have challenged ideas related to the meaning of maleness and femaleness. While sex-role-reversed mating systems are quite rare in most taxonomic groups, sex-role reversal has nevertheless been documented in fishes, amphibians, birds, and insects (reviewed in [5]). Work over the last several decades has elucidated many of the ultimate mechanisms responsible for the evolution of sex-role reversal, such as effects of parental investment on the operational sex ratio, the environmental potential for polygamy, and the Bateman gradient [4,6,7]. However, many of the proximate effects of sex-role reversal are not well understood, even though we know that the evolution of sex-role reversal is accompanied by potentially dramatic changes in morphology, behavior, and reproductive physiology.

The phenotypic manifestations of sex-role reversal include traits such as showy ornaments and increased aggression in females, as well as marked mating preferences in males [8]. In addition, sex-role-reversed species also often have adaptations associated with male parental investment [9]. Many of these obvious external features might be expected to be accompanied by underlying metabolic or physiological changes [5,10]. Because the traits associated with sex-role reversal tend to be sexually dimorphic, their expression should be encoded by genetic pathways that are connected to the sex-determination hierarchy. While little is known about the mechanisms underlying the genetic basis of sexual dimorphism, previous studies have shown that genes on the sex chromosomes and genes regulated by the hypothalamic-pituitary-gonadal axis, such as growth hormone (GH), are often involved in regulating size dimorphisms [11]. In vertebrates, such connections involve sex hormones, so most of the studies aimed at understanding proximate mechanisms regulating sex-role-reversed morphology and behavior have focused on the endocrinology of sex-role reversal, with a particular emphasis on birds [5,12].

The endocrinology of sex-role-reversed species has perhaps generated more questions than answers. One general pattern is that female maturation and cycles of gamete production are controlled by estrogens, whereas male primary sexual traits are controlled by androgens, in much the same way as reproduction is controlled in species with conventional sex roles [5,10]. However, efforts to pin down the proximate causes of female-specific morphology and behavior in sex-role-reversed taxa have revealed a complex picture that appears to vary among taxa. For instance, in most species of sex-role-reversed birds, males have higher testosterone levels than females [13], but there is some indication that testosterone levels nevertheless modulate female aggression, perhaps because certain regions of the female brain are more sensitive to androgens compared to the male brain [14]. On the other hand, in some sex-role-reversed birds, such as barred buttonquails and moorhens, males and females exhibit similar plasma levels of testosterone [15,16], so general patterns regarding testosterone’s role have yet to emerge. In addition, hormones other than testosterone have been implicated in sex-role reversal in birds. For example, progesterone modulates female aggression in the black coucal [17], and males have higher prolactin levels than females in spotted sandpipers [18] and Wilson’s phalarope [19], a reversal of the levels normally observed in species with conventional sex roles. Few studies have been conducted on the endocrinology of non-avian sex-role-reversed species, but what little work has been done indicates a complex picture in these other taxa as well. For instance, in sex-role-reversed populations of the peacock blenny (a marine fish), estradiol and prostaglandin F2α are involved in female courtship displays and mating behavior [20]. In short, the endocrinology of sex-role reversal appears to be complex and remains poorly understood.

One noticeable void in the study of proximate mechanisms of sex-role reversal exists in the realm of transcriptome-level responses of sexually dimorphic tissues to hormonal manipulation. Here we begin to fill this gap by studying the transcriptome of the Gulf pipefish (*Syngnathus scovelli*) liver exposed to the potent endocrine disruptor 17α-ethinylestradiol (EE2). We chose to study the Gulf pipefish because it has become an excellent model for the study of many aspects sex-role reversal, and it is an emerging model in the realm of sex-role-reversed endocrinology [10]. Several important features of the liver motivated us to focus on this organ, rather than other obvious choices such as the gonads or male brood pouch, for this initial analysis of transcriptome-level sexual dimorphism and response to an environmental estrogen. First, unlike testes and ovaries, the liver is present in both males and females, facilitating a meaningful comparison of transcriptomes between the sexes. Second, livers in general have been shown to be sexually dimorphic [21,22,23], and in fish the liver in particular plays a major role in reproduction, as many important egg proteins are produced in the liver and transported to the eggs in females. Third, several important liver genes, such as *vitellogenin* and *zona pellucida* isoforms, are known to be estrogen dependent [24] and thus provide clear expectations regarding changes in expression levels in response to elevated levels of estrogenic compounds.

Studies regarding the effects of environmental estrogens in Gulf pipefish and its close relatives provide context for the present study. In particular, we know that external female morphology is estrogen-dependent, because exposure of male Gulf pipefish to 17α-ethinylestradiol (EE2) results in the development of female-like secondary sexual traits [25,26]. In addition, expression of *vitellogenin* has been shown to be induced by estradiol or EE2 in four pipefish species and a seahorse, *Syngnathus acus*, *S*. *scovelli*, *S*. *abaster*, *Hippocampus guttulatus* (also known as *H*. *ramulosus*) and *Nerophis lumbriciformis* [25,27,28], indicating that this gene is estrogen-regulated in syngnathid fishes. Sufficiently high levels of EE2 (above about 5 ng/L) prevent males from developing a functional brood pouch and consequently cause complete reproductive failure [26,29]. This observation is consistent with other data from unpublished dissertation work, which indicates that the male brood pouch is androgen-dependent (reviewed in [10]). However, thus far no evidence suggests that EE2-exposed males adopt female-typical courtship behavior [28], so the proximate mechanisms of sex-role reversal in pipefish seem to mirror those of avian taxa in terms of complexity. As a first step toward understanding transcriptome-level responses to hormone manipulation in a sex-role-reversed pipefish, we used RNA-sequencing (RNA-seq) to characterize the liver transcriptomes of female, pregnant male, and non-pregnant male Gulf pipefish exposed to the potent endocrine disruptor EE2. Our first goal was to examine patterns of sexual dimorphism in liver gene expression in non-exposed control animals to identify genes displaying sexual dimorphism or responsiveness to male pregnancy status. We hypothesized that genes involved directly in egg production would show much higher levels of expression in females than in males, but we also recognized the possibility that constraints imposed by male pregnancy could result in the production of additional compounds in the livers of either males or females. For example, if females package additional proteins in their eggs to function during post-copulatory processes, potentially occurring within the male’s brood pouch, or if males provision their offspring, then some important proteins involved in these processes could conceivably be produced in the livers of either sex.

Our second goal was to quantify gene expression changes in the liver in response to estrogen exposure in females, pregnant males, and non-pregnant males. We hypothesized that the general pattern of expression changes would be toward feminization of male livers. However, if multiple endocrine signaling pathways have been altered to different degrees by the evolution of sex-role reversal, then we might expect an intermediate pattern, in which some genes are very strongly affected by EE2 while others are relatively resistant even though they are nevertheless sexually dimorphic in their expression patterns. We further assessed whether our observed gene expression patterns are sex-role reversed by comparing our results to those from the zebrafish (*Danio rerio*), a model organism with conventional sex roles.

## Results

### Sexually Dimorphic Gene Expression Patterns

The RNA-seq results identified a total of 482 Trinity gene transcripts that were significantly differentially expressed in the control female livers compared to the livers of control pregnant and non-pregnant males, with a false-discovery-rate corrected *p* ≤ 0.05. Of these 482 differentially expressed transcripts, 67% were up-regulated in females (325 contigs) and the remaining 33% showed higher expression levels in males (157 contigs) (S1 Table). The gene ontology analyses in Blast2GO identified two biological processes, cellular process and metabolic process, as being the most common in the control female liver transcripts. These two processes accounted for 22 and 19 percent, respectively, of the biological processes represented in the annotated sequences (S1 Table).

Of the 325 up-regulated transcripts in females, a total of 21 genes showed an over-expression of 20 fold or higher compared to males (Table 1). These over-expressed genes included several known female-specific genes, such as *vitellogenin b* and *c*, *choriogenin h*, and *zona pellucida sperm-binding protein 4-like*, which have all been shown to be involved in egg development and maturation [30,31,32]. In addition to the female-specific genes, *estrogen receptor alpha* (*esr1*) was also highly up-regulated in control females. Several of the genes with the largest differences in expression levels, such as *extracellular serine threonine kinase fam 20c-like* (*Fam20c*), were labeled as “cellular response to estrogen stimulus” genes in the Blast2GO analysis. Many of these strongly sexually dimorphic genes have also been shown to have female-biased hepatic expression profiles, particularly in response to estrogen exposure [33]. Examples include *3-hydroxy-3-methylglutaryl-coenzyme a reductase* (*hmgcra*), *methylsterol monooxygenase* 1(*msmo1*), and *lanosterol 14-alpha demethylase-like* (*CYP51A1*), in addition to the aforementioned female-specific genes. Of the 157 genes that were up-regulated in males, only one transcript, *histidine triad nucleotide-binding protein 3-like* (*hint3*, S1 Table), exhibited a greater than 20-fold difference in expression levels between males and females.

**Table 1:** Hepatic Genes with Female Biased Expression Patterns in Control Fish Livers.

Top Blastx Hit Description	Fold Change
vitellogenin c	1588
c44657_g1_i1	1579
extracellular serine threonine protein kinase fam20c-like	1207
vitellogenin b	1031
brain-specific angiogenesis inhibitor 1-associated protein 2-like protein 2-like	792
c48786_g1_i1	692
cathepsin e-like	635
choriogenin h	528
extracellular serine threonine protein kinase fam20c-like	458
extracellular serine threonine protein kinase fam20c-like	456
zona pellucida sperm-binding protein 4-like	239
estrogen receptor alpha	47
c55348_g2_i1	40
3-hydroxy-3-methylglutaryl-coenzyme a reductase	39
c38044_g1_i1	34
methylsterol monooxygenase 1	30
sodium- and chloride-dependent creatine transporter 1-like	30
c43284_g1_i1	29
sodium- and chloride-dependent creatine transporter 1-like isoform x1	23
lanosterol 14-alpha demethylase-like	21
monocarboxylate transporter 13-like	20

List of blastx annotation and fold change information for all genes showing female-biased expression patterns with fold changes of 20 or higher in control females relative to control males.

A comparison of the transcripts between the sexes by absolute number of reads showed that 46 of the top 50 expressed genes were shared between the two sexes (Table 2). Females had four genes within their 50 highest expressed transcripts that were not included within the males’ top 50, including transcripts for a translationally controlled tumor protein and three egg proteins: *vitellogenin b*, *zona pellucida sperm-binding protein 4-like*, and *chorion protein*. The males also had four genes that were found solely in the list of 50 top expressed genes for males but not females. These genes included *pancreatic elastase*, *coagulation factor VIIb precursor*, *fibrinogen alpha chain-like*, and a hypothetical unknown protein gene.

**Table 2:** Top 50 Expressed Genes in the Male and Female Livers for Control Fish.

	**Top 50 Expressed Genes in Males**	**Contig ID**	**Mean # reads**
1	warm temperature acclimation-related 65 kDa protein	c16137_g1_i1	313030
2	ApoA-I	c41331_g1_i1	161667
3	TPA: hypothetical protein BOS_23215	c20770_g1_i1	232799
4	PREDICTED: histidine-rich glycoprotein-like	c33611_g1_i1	294179
5	complement component C3	c56882_g1_i1	281272
6	hypothetical protein OXYTRI_13058	c27857_g1_i1	260783
7	14 kDa apolipoprotein, partial	c28061_g4_i1	171653
8	transferrin	c48809_g2_i1	112617
9	PREDICTED: betaine—homocysteine S-methyltransferase 1	c54924_g2_i1	129460
10	cytochrome c oxidase subunit I	c59228_g4_i1	144836
11	astacin like metalloprotease	c11350_g1_i1	99607
12	alpha-1-antitrypsin	c45579_g1_i1	103302
13	PREDICTED: myeloid protein 1-like	c48382_g1_i1	98270
14	alpha-1-antitrypsin	c51148_g1_i1	76306
15	chymotrypsinogen 1	c78479_g1_i1	58669
16	PREDICTED: beta-microseminoprotein-like	c52972_g5_i1	71077
17	complement C1q tumor necrosis factor-related protein 3-like	c43914_g2_i1	73256
18	hyaluronan binding protein 2 precursor	c52158_g2_i1	59368
19	PREDICTED: fibrinogen gamma chain	c47758_g1_i1	63278
20	Trans-1,2-dihydrobenzene-1,2-diol dehydrogenase	c47414_g1_i1	68241
21	PREDICTED: collagenase 3-like	c58007_g5_i1	65271
22	PREDICTED: type-4 ice-structuring protein-like isoform X2	c33291_g1_i1	52243
23	PREDICTED: bile acid receptor-like isoform X2	c50783_g1_i2	49235
24	PREDICTED: inter-alpha-trypsin inhibitor heavy chain H3-like	c56014_g1_i2	58790
25	trypsin-2 precursor	c6350_g1_i1	49044
26	fatty acid-binding protein	c27863_g1_i1	39381
27	aldolase A	c56993_g6_i1	46269
28	hypothetical protein, partial	c42909_g1_i1	44988
29	PREDICTED: chymotrypsin-like protease CTRL-1	c51234_g1_i1	44034
30	PREDICTED: ceruloplasmin-like	c48515_g2_i6	40423
31	PREDICTED: LOW QUALITY PROTEIN: selenoprotein P	c55131_g1_i1	40219
32	PREDICTED: alpha-2-macroglobulin-like isoform X1	c58355_g3_i1	45394
33	PREDICTED: protein AMBP-like	c52079_g1_i1	46994
34	complement regulatory plasma protein	c51045_g2_i1	40653
35	Pancreatic elastase	**c24626_g1_i1**	32525
36	elongation factor 1 alpha	c43004_g1_i1	36091
37	PREDICTED: prothrombin	c47589_g1_i1	35742
38	PREDICTED: complement C5-like	c46369_g3_i1	34227
39	PREDICTED: complement factor B-like	c57046_g1_i1	35738
40	coagulation factor VIIb precursor	**c53536_g1_i1**	37228
41	PREDICTED: fibrinogen beta chain	c50315_g1_i1	20373
42	vitellogenin c	c52176_g1_i1	19005
43	PREDICTED: alpha-2-HS-glycoprotein-like	c41324_g1_i1	29738
44	hypothetical protein, partial	**c55432_g7_i1**	36658
45	PREDICTED: alpha-2-HS-glycoprotein-like	c41324_g1_i1	41955
46	plasminogen	c40786_g1_i1	31122
47	hypothetical protein, partial	c12437_g1_i1	24375
48	glyceraldehyde-3-phosphate dehydrogenase	c44637_g1_i1	25244
49	PREDICTED: fibrinogen alpha chain-like	**c55500_g1_i1**	33769
50	PREDICTED: kininogen-1-like	c54367_g1_i1	25964
	**Top 50 Expressed Gene in Females**	**Contig ID**	**Mean # reads**
1	Ribosomal 40s 18s	c27857_g1_i1	602577
2	vitellogenin b	**c58295_g2_i1**	583908
3	vitellogenin c	c52176_g1_i1	197651
4	TPA: hypothetical protein BOS_23215	c20770_g1_i1	179421
5	PREDICTED: histidine-rich glycoprotein-like	c33611_g1_i1	172732
6	complement component C3	c56882_g1_i1	104763
7	cytochrome c oxidase subunit I	c59228_g4_i1	104364
8	PREDICTED: betaine—homocysteine S-methyltransferase 1	c54924_g2_i1	86711
9	ApoA-I	c41331_g1_i1	80579
10	14 kDa apolipoprotein, partial	c28061_g4_i1	60526
11	alpha-1-antitrypsin	c45579_g1_i1	59001
12	astacin like metalloprotease	c11350_g1_i1	55898
13	warm temperature acclimation-related 65 kDa protein	c16137_g1_i1	53062
14	PREDICTED: beta-microseminoprotein-like	c52972_g5_i1	46663
15	transferrin	c48809_g2_i1	46224
16	PREDICTED: myeloid protein 1-like	c48382_g1_i1	42133
17	aldolase A	c56993_g6_i1	39493
18	Trans-1,2-dihydrobenzene-1,2-diol dehydrogenase	c47414_g1_i1	36096
19	elongation factor 1 alpha	c43004_g1_i1	34786
20	PREDICTED: inter-alpha-trypsin inhibitor heavy chain H3-like	c56014_g1_i2	30860
21	trypsin-2 precursor	c6350_g1_i1	29855
22	fatty acid-binding protein	c27863_g1_i1	29508
23	chymotrypsinogen 1	c78479_g1_i1	29312
24	alpha-1-antitrypsin	c51148_g1_i1	28159
25	PREDICTED: inter-alpha-trypsin inhibitor heavy chain H3-like	**c58914_g1_i1**	27712
26	hyaluronan binding protein 2 precursor	c52158_g2_i1	27393
27	complement C1q tumor necrosis factor-related protein 3-like	c43914_g2_i1	27226
28	PREDICTED: fibrinogen gamma chain	c47758_g1_i1	26773
29	PREDICTED: alpha-2-HS-glycoprotein-like	c41324_g1_i1	25675
30	zona pellucida protein sperm-binding protein 4-like	**c47764_g1_i1**	23924
31	PREDICTED: bile acid receptor-like isoform X2	c50783_g1_i2	23763
32	PREDICTED: collagenase 3-like	c58007_g5_i1	21668
33	PREDICTED: type-4 ice-structuring protein-like isoform X2]	c33291_g1_i1	21626
34	PREDICTED: LOW QUALITY PROTEIN: selenoprotein P	c55131_g1_i1	21530
35	complement regulatory plasma protein	c51045_g2_i1	20297
36	chorion protein	**c41859_g1_i1**	18712
37	PREDICTED: chymotrypsin-like protease CTRL-1	c51234_g1_i1	18248
38	PREDICTED: ceruloplasmin-like	c48515_g2_i6	18197
39	PREDICTED: fibrinogen beta chain	c50315_g1_i1	17399
40	glyceraldehyde-3-phosphate dehydrogenase	c44637_g1_i1	17202
41	PREDICTED: protein AMBP-like	c52079_g1_i1	17066
42	PREDICTED: complement factor B-like	c57046_g1_i1	16969
43	PREDICTED: kininogen-1-like	c54367_g1_i1	16600
44	translationally-controlled tumor protein	**c38295_g1_i1**	15892
45	hypothetical protein, partial	c42909_g1_i1	15834
46	plasminogen	c40786_g1_i1	15555
47	PREDICTED: prothrombin	c47589_g1_i1	15421
48	PREDICTED: alpha-2-macroglobulin-like isoform X1	c58355_g3_i1	15328
49	PREDICTED: complement C5-like	c46369_g3_i1	14994
50	hypothetical protein, partial	c12437_g1_i1	14889

The top blastx hit descriptions, contig numbers, and mean number of reads for control males and control females are shown. The bold contig IDs represent the four sex-specific genes for each sex.

**Fig 1:**
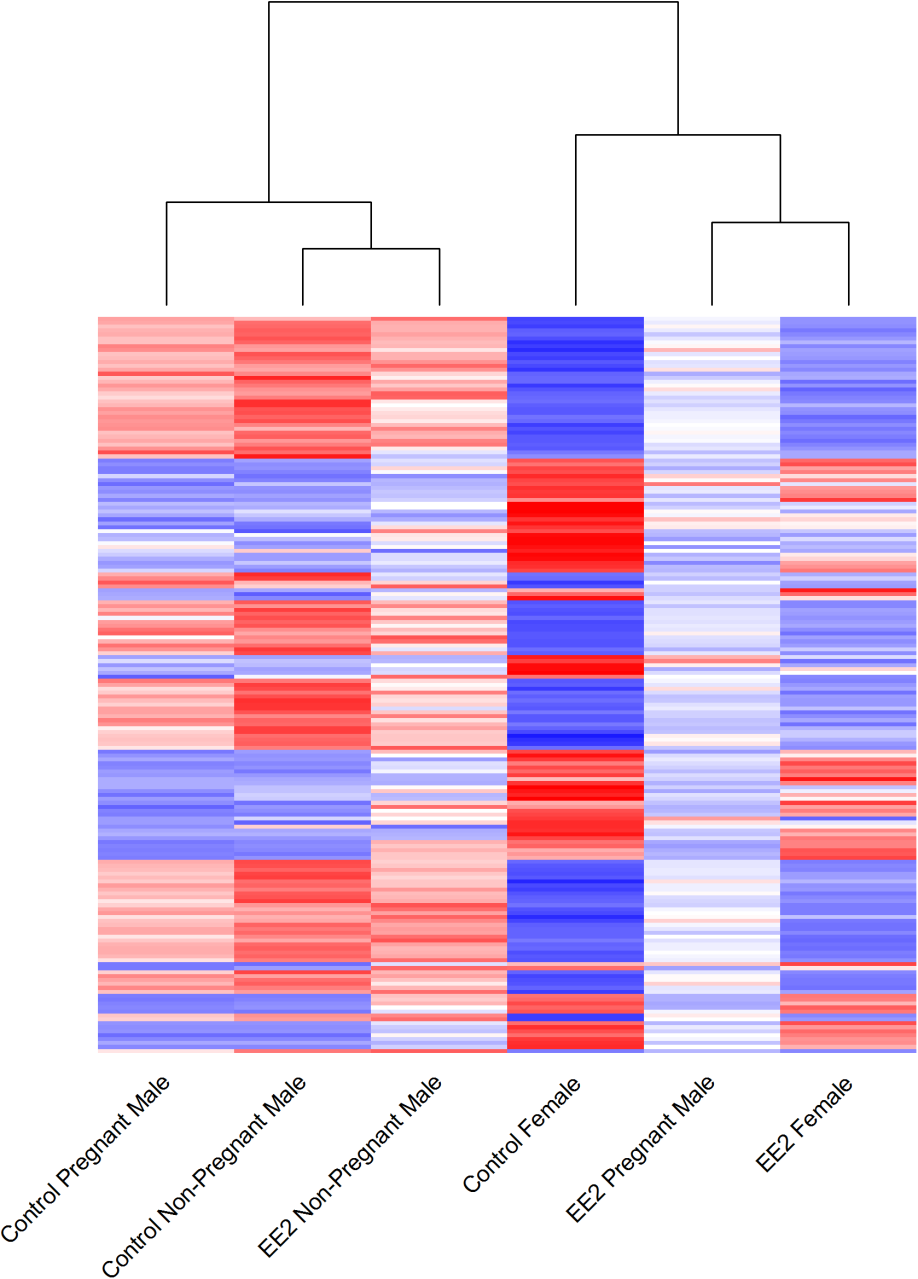
Sexually Dimorphic Gene Expression Patterns. Heat map showing hierarchical clustering of mean expression values for the six treatments including control females, pregnant males, and non-pregnant males, as well as EE2 exposed females, pregnant males, and non-pregnant males, for all genes which showed sexually dimorphic expression patterns in control fish. The colors of the bars represent either up-regulated (red) or down-regulated (blue) genes. The male treatments cluster together with the exception of the EE2 exposed pregnant males which cluster with the both control and EE2 females.

Restricting attention to sexually dimorphic genes, we found that control pregnant males and non-pregnant males had very similar patterns of gene expression to one another that were quite distinct from those of females (Fig 1). This observation supports the hypothesis that both pregnant and non-pregnant males have a male-like gene expression pattern that differs drastically from that of females. A comparison of gene expression between control pregnant and control non-pregnant males showed that 41 genes were differentially expressed between these categories of males. Of these 41 transcripts, two genes (contig c58371_g3_i3 and *cornifelin homolog b-like*) were upregulated by greater than 20 fold in pregnant males, and one gene (*cartilage acidic acid 2*) was upregulated greater than 20-fold in non-pregnant males (S2 Table).

### Effects of Synthetic Estrogen Exposure

**Fig 2:**
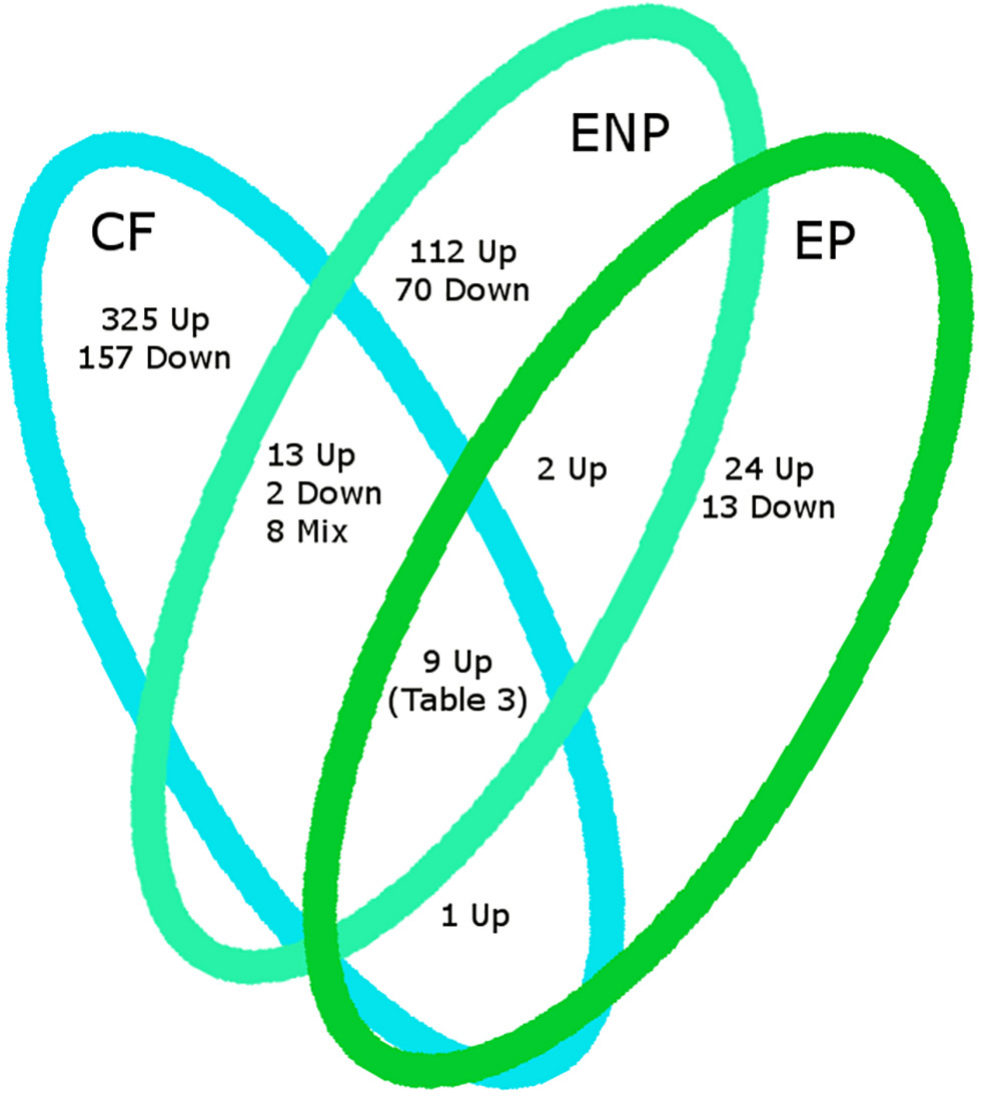
The Overlap of Female Biased and EE2 Responsive Genes. Venn diagram of control females (CF), EE2 exposed pregnant males (EP), and non-pregnant males (ENP) to show overlap of female biased genes and EE2 responsive genes. Gene expression levels for control females are either over or under expressed compared to the levels of expression in control males, and EE2 exposed male genes were differentially expressed from their control counterparts. A list of genes up-regulated between all three groups can be found in Table 3.

After one week of exposure to 5ng/L EE2, both the pregnant and non-pregnant males’ gene expression patterns demonstrated a clear response to estrogen and showed signs of feminization (Fig 1). Non-pregnant males exhibited a greater response to EE2 exposure than did pregnant males, with a total of 182 genes differentially expressed (112 up-regulated and 70 down-regulated) in EE2-exposed non-pregnant males relative to control non-pregnant males (Fig 2, S3 Table). We detected only 37 differentially expressed gene transcripts in exposed pregnant males relative to control pregnant males (24 up-regulated and 13 down-regulated; Fig 2, S4 Table). Two of the genes up-regulated in exposed non-pregnant males, but not in exposed pregnant males, were contig c58371_g3_i3 and *cornifelin homolog b-like*, which also were up-regulated in control pregnant males (S3 Table, S4 Table). A subset of nine genes was up-regulated in control females (relative to control males), EE2-exposed pregnant males (compared to control pregnant males) and EE2-exposed non-pregnant males (relative to control non-pregnant males). These loci included *cathepsin e-like* (a liver-specific aspartic proteinase), *extracellular serine threonine protein kinase fam20c-like*, and *choriogenin h*, among others (Table 3; Fig 2). Among the subset of nine genes showing both female biased expression and EE2 responsiveness in exposed males, nearly half of these genes were known egg-associated loci, such as *vitellogenin b* and *c*, *choriogenin h* and *l*, and *zona pellucida sperm-binding protein 4-like* (Table 3), which are normally expressed at high levels in females only. A comparison of exposed pregnant males to exposed non-pregnant males revealed one gene that was upregulated greater than 20-fold in pregnant males (contig c59977_g1_i1) and two genes that were upregulated greater than 20-fold in non-pregnant males (*enolase-phosphatase E1-like* and *fibrous sheath CABYR-binding protein-like*; S5 Table).

**Table 3:** Genes Showing Both Female Biased Expression in Control Fish and EE2 Responsiveness in Exposed Males.

Top Blastx Hit Descriptions	Control Female	EE2 Preg Male	EE2 Non-Preg Male
Upregulated compared to:	Control Males	Control Preg Males	Control Non-Preg Males
vitellogenin c	1587	291	62
choriogenin h	528	67	94
zona pellucida sperm-binding protein 4-like	239	157	83
extracellular serine threonine protein kinase fam20c-like	1207	125	268
brain-specific angiogenesis inhibitor 1-associated protein 2-like	792	66	70
cathepsin e-like	635	141	451
sodium- and chloride-dependent creatine transporter 1-like	30	13	6
protein jagunal homolog 1-b-like	1.2	1.1	1.3
c48786_g1_i1	691	225	139

List of shared, up-regulated genes in control females (when compared with control males) and EE2 exposed pregnant and non-pregnant males (when compared their control counterparts) and their significant fold inductions.

The livers of EE2-exposed females did not show a pattern of increased feminization relative to livers of control females. Rather, we saw expression patterns respond in the opposite direction. A total of 20 genes were found to be sexually dimorphic in control fish and also differentially expressed between EE2-exposed and control females (Table 4). Eighteen of these 20 transcripts showed a pattern in which the effects of EE2 exposure in female livers were of opposite sign compared to the pattern of sexual dimorphism. For instance, genes that were upregulated in female control fish relative to male controls were usually downregulated in EE2-exposed females relative to control females. However, these effects were relatively small (Table 4), and no transcripts showed a greater than 20-fold expression difference in females as a result of EE2 exposure (S6 Table).

**Table 4:** Expression Patterns for Female EE2 Responsive Genes.

Top Blastx Hit Descriptions	Fold Change for Control Females	Fold Change for EE2 Exposed Females
adenylosuccinate synthetase isozyme 1 c-like	1.6	-1.9
cytosolic carboxypeptidase-like protein 5-like isoform x1	1.5	-1.7
40s ribosomal protein s18	2.3	-5.4
myelin protein zero-like protein 3-like	1.2	-1.7
lysosomal acid phosphatase precursor	1.7	-5.0
rna-directed dna polymerase from mobile element jockey-like	1.4	-3.2
warm temperature acclimation protein 65–2	1.7	-7.7
26s protease regulatory subunit 7	-2.3	1.7
angiotensinogen	-3.8	1.7
atpase asna1	-2.9	1.8
camp-regulated phosphoprotein 19	-2.6	1.4
cytochrome c	-4.1	1.8
mitochondrial rho gtpase 2-like	-3.6	1.6
nadh dehydrogenase	-2.4	2.0
peptidyl-prolyl cis-trans isomerase h	-3.3	2.2
proteasome subunit alpha type-3	-2.6	1.7
proteasome subunit beta type-4-like	-3.3	1.7
threonine—trna cytoplasmic	-8.2	2.7
run and fyve domain-containing protein 2-like isoform x1	-1.1	-1.7
solute carrier family facilitated glucose transporter	-4.2	-5.8

List of blastx annotation and fold change information for all genes showing female-biased expression patterns in control females and EE2-responsive patterns in EE2-exposed females when compared with control females.

A principal components analysis (S1 Fig) shows a clear separation of control males and females, regardless of female exposure status. The top loadings for PC1, which explains 49-percent of the variation, include *vitellogenin b*, *warm temperature acclimation-related 65 kDa protein*, and *ribosomal protein 40s 18s*, indicating that PC1 captures variation in some of the genes with the largest sex biases in expression. Exposed pregnant males fall between the females and the rest of the males (S1 Fig). Overall, the first three principal components account for 85 percent of the variation, with three of the top genes that represent the highest loadings in PC1 also listing in the top loadings for PC2 and PC3. Restricting attention to the sexually dimorphic genes, we see a general trend for males to become feminized in their gene expression patterns as a result of EE2 exposure (Fig 1). The grouping of EE2-exposed pregnant males with females in Fig 1 and S1 Fig indicates that exposure to synthetic estrogen makes pregnant male livers more female-like than it does to non-pregnant male livers. However, this observation should be interpreted in light of our previous result that EE2 affects the expression patterns of more genes in non-pregnant male livers relative to pregnant male livers.

### Comparison to Zebrafish

**Fig 3:**
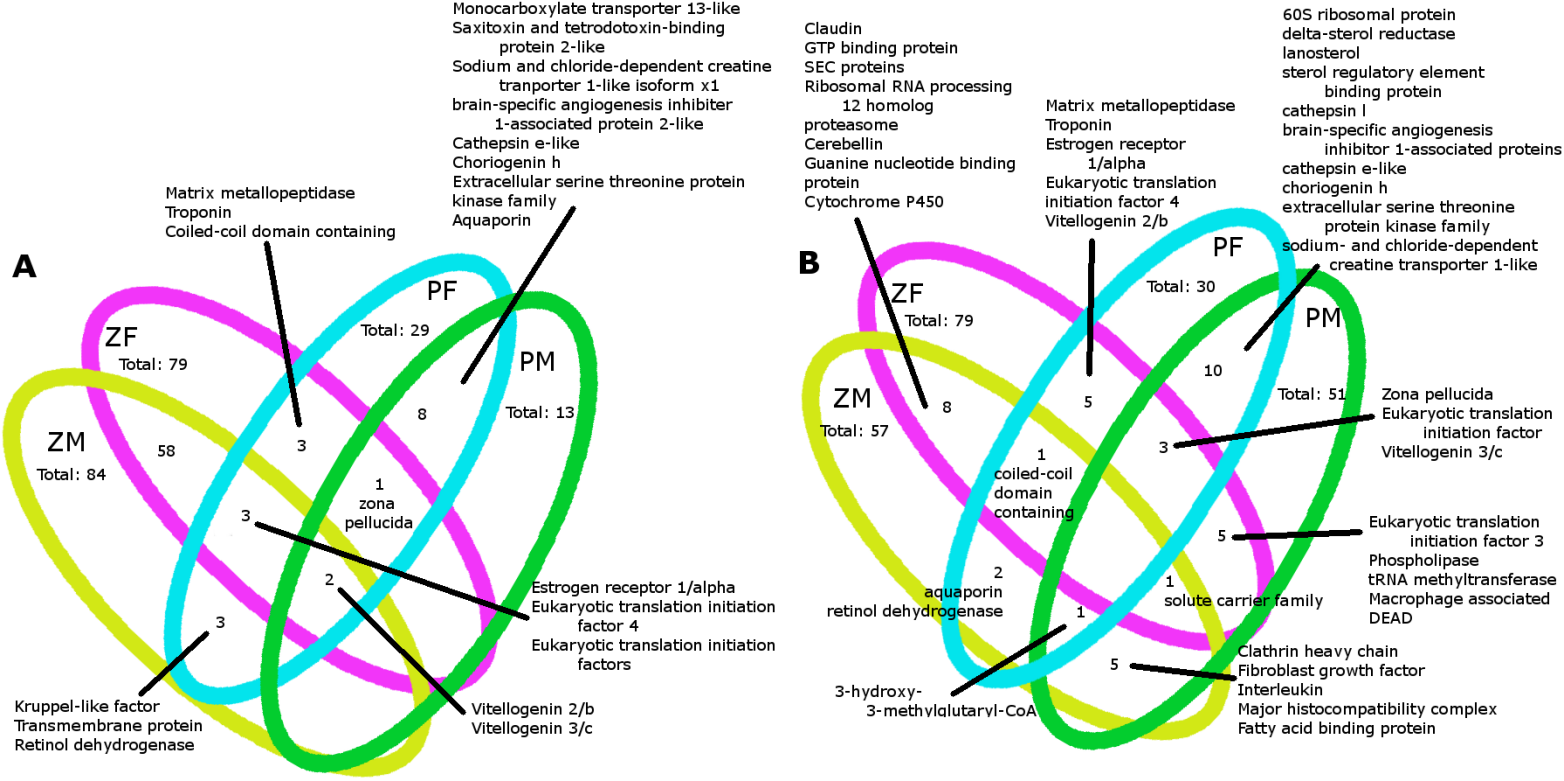
Pipefish and Zebrafish Comparisons of Expression Patterns. Venn diagrams of sexually dimorphic and EE2 responsive genes for pipefish and zebrafish showing EE2 up-regulated genes in 3a and EE2 down-regulated genes in 3b. Numbers of female biased genes in control females are presented for control pipefish females in this study (PF) and for control zebrafish from a study by [23]. (ZF). Male EE2 responsive genes are presented for both pregnant and non-pregnant, EE2 exposed pipefish males (PM) and for EE2 exposed males from [23](ZM).

We found that the majority of the sex-biased genes in zebrafish and pipefish did not overlap, with only nine female-biased genes shared between non-exposed females of the two species (Fig 3A). In the comparison of the down-regulated genes in females, only four were shared between non-exposed zebrafish and pipefish (Fig 3B). Most of the genes that were up-regulated in males of either species in response to EE2 exposure were also female-biased genes (63 of 84 genes in zebrafish males and 11 of 13 genes with 20-fold or higher expression changes in pipefish). The only genes that were female-biased in both species and up-regulated in EE2-exposed males of both species were *vitellogenin 2/vitellogenin b* and *vitellogenin3/vitellogenin c*. When only sexually-dimorphic genes were considered, pipefish males had more genes that were down-regulated than up-regulated in response to EE2 exposure, which was the opposite pattern observed in zebrafish males. Although some of the genes that were down-regulated as a result of EE2 exposure were female-biased (14 of 51 in pipefish and 10 of 57 in zebrafish), the majority were not. In fact, most EE2 responsive, down-regulated genes in zebrafish were male-biased (38 of 57). However, in pipefish, only 11 of 51 down-regulated genes were male-biased.

## Discussion

This first study of the effects of a potent endocrine disruptor on gene expression patterns in a sex-role-reversed organism shows that the liver of the Gulf pipefish is markedly sexually dimorphic and responds very strongly to an estrogenic compound. For instance, several female-biased genes respond to EE2 exposure in males in a direction indicative of feminization of the male liver transcriptome. Control fish follow a pattern predicted by the hypothesis that both pregnant and non-pregnant males should differ from females in terms of expression patterns as a consequence of the major role of the liver in producing egg-related proteins. Our results also show that fish exposed to synthetic estrogen show expression patterns consistent with the idea that, even in sex-role-reversed species, female-biased liver genes should be regulated by estrogen. In particular, we see a shift toward more feminine patterns of gene expression in males exposed to EE2. Some aspects of our analysis raise the intriguing possibility that the liver plays a role in not only female reproduction but also male reproduction, as sperm-related transcripts occur in the liver, an observation that leads to the hypothesis that males may also be capable of transporting proteins from the liver to the brood pouch. In addition, as we will explain below, we believe our results leave the door open for sexual selection to act on genes expressed in the liver that ultimately play a role in postcopulatory sexual selection or sexual conflict.

### The Endocrinology of Sex-Role Reversal

Sex-role-reversed organisms represent an evolutionary challenge from an endocrinology perspective [5,10], because the females must evolve nuptial ornaments and competitive mating behavior, possibly including enhanced aggression, while simultaneously preserving female reproductive cycles and primary sexual traits. All of these traits related to reproduction and mating competition are potentially sexually dimorphic and hormonally regulated. All sex-role-reversed vertebrates evolved from ancestral taxa with conventional sex roles, and in such taxa androgens (e.g., testosterone or 11-ketotestosterone) are the main hormones involved in almost every aspect of male reproductive competition from the development of primary sexual traits to the modulation of secondary sexual traits and mating behavior [34,35]. Consequently, a male can conceivably become more ornamented or more aggressive during contests for mates by increasing testosterone levels without impairing the male’s ability to produce gametes, although testosterone does have other costs [36]. In sex-role-reversed species, in contrast, a female competing for mates cannot increase her competitive ability simply by increasing testosterone levels without suppressing normal female reproduction, so the evolution of sex-role reversal must have involved mechanisms to circumvent this constraint [5,10]. The diagnosis of these mechanisms has been difficult and remains a work in progress, so we discuss in turn three aspects of female reproduction, namely reproductive physiology, female ornamentation, and female mating behavior, in light of our results and the broader literature on the endocrinology of sex-role reversal.

Our study is most relevant to female reproductive physiology and indeed represents the first dataset addressing transcriptome-wide effects of hormone manipulation in any reproduction-related tissue from a sex-role-reversed species. Our results provide the first evidence that the pipefish liver shows genome-wide patterns of dimorphism, with hundreds of genes differentially expressed between males and females. Some transcripts, such as *vitellogenin b* and *vitellogenin c*, showed greater than 1000-fold higher levels of expression in females compared to males. Our EE2 results establish that these expression patterns are largely estrogen-regulated, because males exposed to EE2 expressed genes that were normally only found at high levels in females. Estrogen profiles have been studied in other sex-role-reversed taxa, notably birds, and in general results show that estradiol profiles are similar to non-sex-role-reversed birds [37,38,39]. The emerging pattern is that estrogens control reproductive physiology in females of sex-role-reversed species in much the same way as in females of species with conventional sex roles. Hence, our data are consistent with an emerging pattern that many sexually dimorphic traits are regulated by the hypothalamic-gonadal-pituitary axis [11].

The situation becomes more confusing when we turn attention to ornamentation in sex-role-reversed taxa. In pipefish, female secondary sexual traits occur in males exposed to EE2, suggesting that female ornamentation is controlled by estrogens [25,26,28]. However, the pattern is markedly different in sex-role-reversed birds. In Wilson’s phalaropes, moorhens and barred buttonquails, injection of females with testosterone increases the brightness of their ornamentation [15,16,40]. Thus, secondary sexual characteristics in these species seem to be affected more by androgens than by estrogens. In other sex-role-reversed marine fishes, such as the peacock blenny and the two-spotted goby, neither estrogens nor androgens seem to be responsible for female nuptial coloration, suggesting an altogether different mechanism could be at work [20,41]). In short, sex-role-reversed females seem to have evolved many disparate mechanisms to modulate nuptial coloration, and pipefish may provide a particularly tractable model because EE2 exposure alone is sufficient to produce female-specific secondary sexual traits in males.

Perhaps the most interesting and demanding area of research in the realm of sex-role-reversed endocrinology concerns the modulation of female behaviors involved in mating competition, and researchers have thus far only scratched the surface of this difficult topic. Almost no work has addressed this topic in the family Syngnathidae, but one study [28] and our anecdotal observations indicate that EE2 exposure does not cause males to adopt female-specific courtship behavior, which in Gulf pipefish includes a striking change in coloration, dancing and twitching [42]. Thus, the proximate factors involved in sex-specific mating behaviors in syngnathids remain unknown. As noted above (see Introduction), the waters are equally muddy for other sex-role-reversed taxa. In particular, testosterone, progesterone, estradiol, and prostaglandins have possible roles in modulating female behavior in various sex-role-reversed taxa [5,17,20]. The endocrinology of mating behavior in sex-role-reversed taxa should be a high priority for research, as many interesting surprises no doubt await us, and syngnathid fishes provide a model uniquely suited to this research endeavor.

### The Pipefish Liver Transcriptome

In many ways, the Gulf pipefish liver transcriptome behaves in a similar fashion to those of the few other fish species studied in this regard. In control fish, pregnant and non-pregnant males shared more similar gene expression patterns than either group shared with females, and both groups of males responded in a female-like manner to synthetic estrogen. The most telling result is that genes involved in egg production are highly upregulated in females relative to males, a typical pattern in the livers of fish species with conventional sex roles. In addition, some of the most highly expressed genes in the livers of females also encode egg-related proteins and EE2 exposure induces expression of these genes in males.

However, at face value, our patterns of gene expression look quite different from those observed in zebrafish [23], with only a few genes occurring on both species’ top-50 lists with regard to expression levels. Similarly, only a handful of genes show similar, statistically significant responses to EE2 in both species. Nevertheless, the overall theme that egg-related protein genes are upregulated in females and that estrogen exposure feminizes male livers appears in both the Gulf pipefish and zebrafish datasets [23]. In addition, the zebrafish study identified a marked up-regulation of ribosomal genes in female livers, and while these genes did not appear on our list of extremely sexually dimorphic loci, many ribosomal genes did appear on our complete list of statistically significant sexually dimorphic genes (S1 Table). Many of the differences between our study and the zebrafish study could be a consequence of differences in experimental design. The zebrafish study used E2 rather than EE2, used a much higher concentration of sex hormones (1000-fold higher), pooled individuals within a treatment before sequencing, and provided some evidence that their males had already been exposed to endocrine disruptors before their use in the experiment [23]. Other studies involving zebrafish [43], yellow perch [44], and turbot [45] show patterns that are broadly consistent with our results, although the precise genes involved often differ considerably among datasets.

Another intriguing result from our analysis is that the low doses of EE2 used in our study resulted in masculinization of the female liver. This pattern is evident from our observation that 90 percent of sexually dimorphic genes showed expression changes in opposition to the pattern of sexual dimorphism in exposed females (i.e., their expression became more male-like). At face value, this observation might be interpreted as evidence that the female liver transcriptome is sex-role-reversed with respect to its response to estrogens. However, this sort of pattern has been observed in other taxa without sex-role reversal [33,46], and the most likely explanation is simply that too much estrogen causes females to begin to shut down reproduction. Thus, this observation also seems to indicate that the pipefish liver is very much like that of other fish without sex-role reversal.

### Sexual Selection and the Liver

One interesting possibility, which is hinted at by our data but not definitively supported by any of our analyses, is that the liver somehow plays a role in sexual selection in sex-role-reversed pipefish. The female liver produces egg-related proteins, which are transported to the ovaries, processed and packaged in the eggs. In sex-role-reversed species, females typically have greater potential reproductive rates than males, which can result in pre- and postcopulatory competition among females for mating and reproductive success. Hence, female-specific genes in the liver controlling egg production could be under stronger sexual selection in females of sex-role-reversed species compared to related species with less mating competition among females, such as monogamous pipefishes and seahorses. Another plausible hypothesis is that other proteins produced by the liver are also transported to the female’s reproductive tract and eventually transferred to the male during mating. Even though most studies of reproductive protein evolution focus on the gonads, the liver may be a neglected organ with an underappreciated role in postcopulatory processes. Interestingly, our data also show that some sperm-related proteins in males are produced in the liver, so males also could experience selection on genes expressed in the liver whose products are ultimately transported to the gonads or brood pouch. This idea remains speculative at the moment, but we feel that the role of the liver in postcopulatory processes, especially in sex-role-reversed organisms, is a topic that deserves further exploration.

### Environmental Endocrine Disruptors in Pipefish

Syngnathid fishes are potentially useful for the study of endocrine disruptors in marine environments, because many pipefishes and seahorses occur in shallow coastal waters throughout the world [47]. Furthermore, many syngnathids, including the Gulf pipefish, lack a pelagic dispersal phase, so most individuals probably complete their lifecycle near their birthplace. Thus, pipefish have the potential to serve as sentinel species for endocrine disruptors in the marine realm. Other work has shown that *vitellogenin* is upregulated in EE2-exposed pipefish males [25,28]. Our results provide a number of other potential genes that could serve as biomarkers of environmental estrogens in male or female pipefish, although males seem to be the best choice for unambiguous evidence of endocrine disruption. One key feature of our study is that we used environmentally relevant concentrations of EE2 (5 ng/L). Studies of EE2 in contaminated bodies of water have revealed levels of EE2 in the range of 30–50 ng/L and even as high as 820 ng/L in some places [48,49,50]. Hence, gene expression changes in pipefish livers could provide a sensitive method for the preliminary screening of sites with a high risk of deleterious impacts from endocrine-disrupting contaminants.

### Conclusions

Our results provide an important advance in the endocrinology of sex-role-reversed vertebrates. In particular we found that the pipefish liver is sexually dimorphic and estrogen regulated, suggesting that female reproductive physiology is regulated by estrogens in pipefish in much the same way as in fish without sex-role reversal. In light of other work on sex-role-reversed taxa, primarily birds, this result suggests that the ancestral mechanism of hormonal control of female reproductive physiology has been retained across sex-role-reversed vertebrates. However, a survey of the literature shows that secondary sexual traits are controlled by a variety of mechanisms, and pipefish are somewhat unusual in that estrogens induce female ornaments in males. Finally, sex-role-reversed mating behaviors remain a mystery, as their proximate modulation has not yet been resolved in any sex-role-reversed taxon. Our results also provide novel pipefish transcripts that respond to endocrine disruptors, which could be extremely useful for screening coastal marine habitats for environmental estrogens. In short, this study provides novel insights into sexual dimorphism and hormonal regulation of gene expression patterns in the pipefish liver, setting the stage for future studies regarding the endocrinology of sex-role-reversed mating behavior in this interesting marine fish.

## Methods

### Experimental Design and Sequencing

Gulf pipefish were collected from Redfish Bay near Aransas Pass, Texas (N 27 53 39.07, W 97 7 51.69) on July 8^th^, 2013 under the Texas Parks and Wildlife permit number SPR-0808-307. This study was approved by the Institutional Animal Care and Use Committee at Texas A&M University (Animal Use Protocol # 2013–0020, Reference #001898) and fish were killed using an overdose of MS-222. We collected only pregnant males and females with well-developed iridescent bands, ensuring that all individuals were sexually mature. Males were allowed to give birth before being used in the experiment. Fish were dipped in freshwater for ten minutes to remove any external parasites and then acclimated to 26-ppt salinity tanks at Texas A&M University. The 17α-ethinylestradiol powder, of 98% purity, was acquired from Sigma (#SLBF2546V) and dissolved in 200-proof ethanol from Sigma (#SHBD6226V). All tanks held 7 liters of water and were initially dosed with 50 μl of either a 7ng/10μl stock EE2 ethanol solution to have tank concentrations of 5ng/L EE2 or 50 μl of ethanol without EE2 for the controls. The chosen concentration of 5 ng/L EE2 was selected because it is within the range of EE2 found in contaminated bodies of water in nature and also because long-term exposure to this concentration has been shown to cause entire breeding populations of some fish species to collapse [49,51,52]. Ten percent water changes were completed daily to ensure that EE2 concentrations remained at a constant 5ng/L as established by [26].

To generate each pregnant male, we placed one non-pregnant male and one female together in a clean, EE2-free saltwater tank until the male became pregnant. Each pair was also assigned a non-pregnant male, size matched with the pregnant male but not paired with a female. On the second day of the male’s pregnancy, all three fish were photographed, measured, and randomly assigned to treatments of either experimental tanks of 5ng/L EE2 concentrations or the EE2-free control tanks on the second day of pregnancy. The three fish were held in 7L tanks with glass dividers between the fish that allowed for water exchange but otherwise kept the fish isolated from one another. After seven days of exposure, that is, on the ninth day of the male’s pregnancy, fish were sacrificed using MS-222 and the liver was dissected. Dissections were done with the fish soaked in RNAlater under a microscope with tissue immediately frozen on dry ice and stored in the -80°C freezer. The control and 5ng/L EE2 treatments were replicated 5 times resulting in a total of 30 fish, including 5 females, 5 pregnant males, and 5 non-pregnant males from each treatment (i.e., EE2 and control).

RNA was isolated from the dissected livers using a TRIzol® Reagent (Life Technologies, Carlsbad, CA) extraction method modified from [53]. Total RNA was sent to the Michigan State University RTSF Genomics Core where libraries were prepared using the TruSeq mRNA Library Prep Kit v2. Libraries were tested for quality control using Caliper GX and qPCR methods. All 30 individuals were barcoded, allowing for each individual’s sequence data to be recovered. The 30 libraries were sequenced using two lanes of an Illumina HiSeq 2500 Rapid Run flow cell v1. Base calling was done by Illumina Real Time Analysis (RTA) v1.17.21.3 and output of RTA was demultiplexed and converted to FastQ with Illumina Bcl2fastq v1.8.4.

### Transcriptome Assembly

A total of 805 million 150 bp paired end reads were obtained from the MSU Genomics core and were then trimmed with Trimmomatic [54] using the following settings: HEADCROP:12, LEADING:10, TRAILING:10, SLIDINGWINDOW: 4:15, MINLEN: 50. All of the paired, trimmed files were next run through the program FLASH to identify and collapse overlapping reads before starting the assembly [55]. All of these prepared reads from the 30 individuals were assembled into a single transcriptome with Trinity using version trinityrnaseq_r20140717 and the default parameters [56]. The Trinity assembly consisted of a total of 174,578 Trinity transcripts and 130,728 Trinity ‘genes’, referred to as contigs throughout the paper. The Trinity assembly had an N50 of 1866, an average contig size of 973 nucleotides, and a total of 169,973,948 assembled bases. From the Trinity assembly, the contig with the longest open reading frame (ORF) per ‘gene’ was retained in the final transcriptome. Rsem was used to map the paired, trimmed reads from all of the individuals to the retained contigs, hereafter referred to as the “liver transcriptome” [57]. The contig counts (FPKM) from Rsem were then analyzed using EBSeq to identify differentially expressed contigs across the treatments and categories within the treatments [58]. Finally, the contigs reported by EBSeq to be differentially expressed were blasted against the non-redundant protein database at NCBI. We also used Blast2Go to ascertain Gene Ontology (GO) functions [59]. To assess the completeness of our transcriptome, we used the program CEGMAv2.5, Core Eukaryotic Gene Mapping Approach, and recovered 244 complete sequences and 247 partial gene sequences from the CEGMA database containing 248 core eukaryotic genes, using a default e-value of 10 [60]. From these CEGMA results, we can conclude that our liver transcriptome has a very high level of completeness, because the majority of the conserved core eukaryotic genes are represented. This CEGMA analysis was employed only to assess the completeness of our transcriptome assembly; all other analyses used the full set of 130,728 Trinity ‘genes’.

### Analysis

After assessing differential expression in all pairwise treatment comparisons, we identified genes that had at least a 2-fold expression difference between control females and control males. These genes were considered to be sexually dimorphic, and their expression patterns were analyzed in all groups, including EE2-exposed fish, using heat maps. Heat maps were generated in R [61] using the package gplots [62]. We also utilized a principal components analysis (PCA) using prcomp in R (R Core Team) to compare gene expression patterns across the treatments. For this analysis, we removed all contigs that had less than the mean number of reads mapped to them (3,090 reads) across the 30 fish from the two treatments.

To address whether the expression patterns we found showed signs of sex-role reversal, we compared our results to those from a similar study in zebrafish [23], a species with conventional sex roles. The zebrafish study exposed males and females to water containing 5 μg/L 17β-estradiol (i.e., a 1000-fold higher concentration than we used in our study) for 48 hours and investigated transcriptome expression in the liver. We were interested in whether female pipefish have similar gene expression patterns to zebrafish females, and whether males exposed to estrogens respond similarly in species with conventional sex roles (zebrafish) and reversed sex roles (pipefish). Venn diagrams were created to compare the number of genes with female-biased expression patterns in control females and the number of up-regulated or down-regulated genes in estrogen-exposed males. To carry out the comparisons between species, gene names were generalized by removing subunit isoform types and by grouping some gene families.

## Supporting Information

S1 Fig A PCA for all six treatments using all genes with a mean number of transcripts equal or greater than 3,090. Although the treatments of the same sex group together, EE2 exposed pregnant males cluster closer to the female treatments than other male groups.
(TIFF)

S1 Table Full list of differentially expressed genes between control females and control males. (XLSX)

S2 Table Full list of differentially expressed genes between control pregnant males and control non-pregnant males. (XLSX)

S3 Table Full list of differentially expressed genes for EE2 exposed pregnant males versus control pregnant males. (XLSX)

S4 Table Full list of differentially expressed genes for EE2 exposed non-pregnant males versus control non-pregnant males. (XLSX)

S5 Table Full list of differentially expressed genes for EE2 exposed pregnant males versus EE2 exposed non-pregnant males. (XLSX)

S6 Table Full list of differentially expressed genes for EE2 exposed females versus control females. (XLSX)
