# Supplementary figures and images for: The Effects of Synthetic Estrogen Exposure on the Sexually Dimorphic Liver Transcriptome of the Sex-Role-Reversed Gulf Pipefish
Source: PLoS One. 2015 Oct 8;10(10):e0139401. doi: 10.1371/journal.pone.0139401 (PMC4598134; doi:10.1371/journal.pone.0139401)

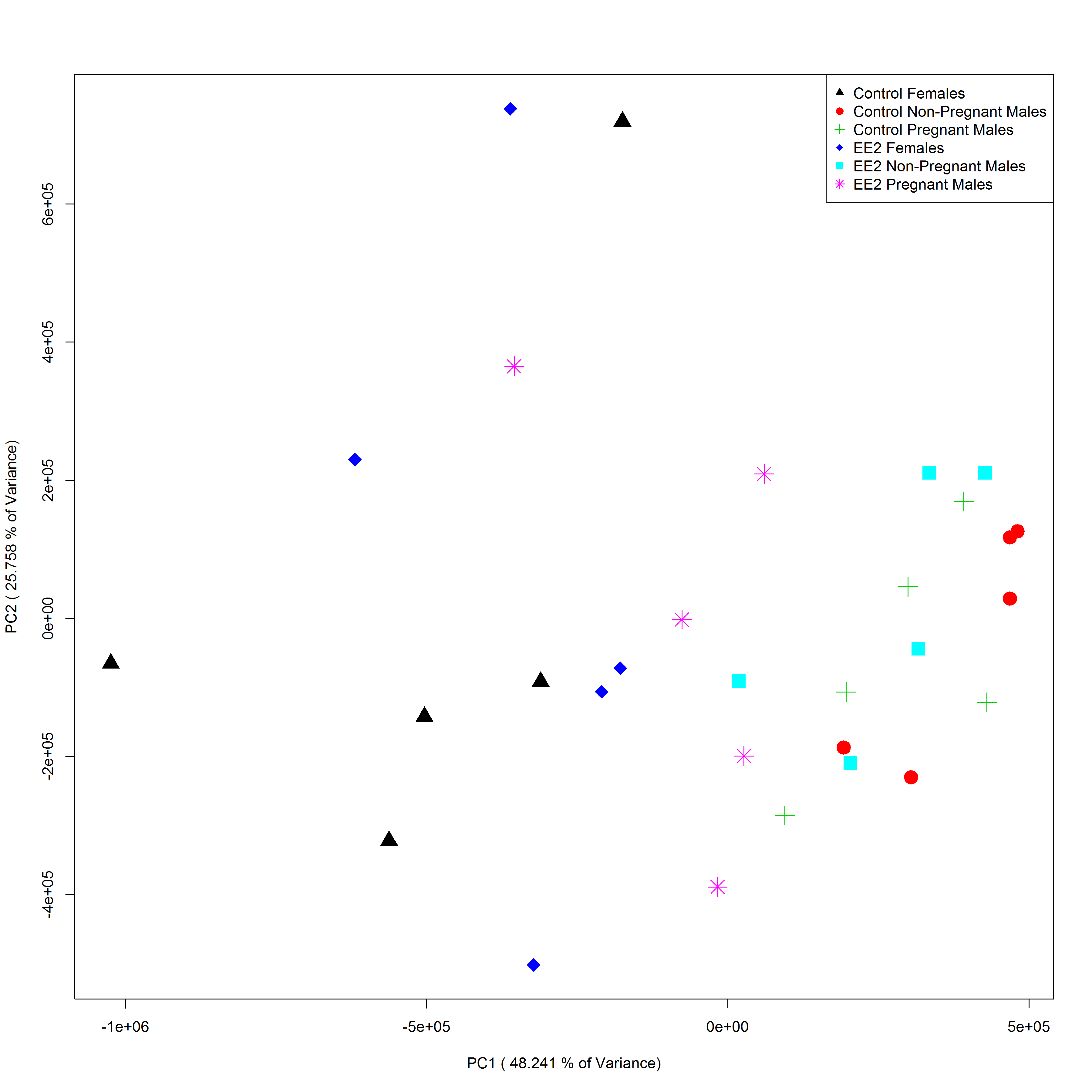

Supplement: S1 Fig — Although the treatments of the same sex group together, EE2 exposed pregnant males cluster closer to the female treatments than other male groups. (TIFF) [file pone.0139401.s001.tiff]
